# Supplementary material for: Are carbon emissions trading and green financial instruments synergistic? -Comprehensive quantitative research based on content analysis
Source: PLoS One. 2024 Mar 7;19(3):e0298601. doi: 10.1371/journal.pone.0298601 (PMC10919601; doi:10.1371/journal.pone.0298601)
Supplement: S1 Data — (ZIP) [file pone.0298601.s001.zip › Data/地级市Z_F工作报告.zip!/╣π╢1⁄2/╔╪╣╪╩╨/└┤╫╘╣1⁄2╓┌║┼í╛┬φ┐╦╩2╛▌═°í┐_2011.docx]

各位代表：

我代表市人民政府，向大会作工作报告，请予审议，并请各位政协委员和其他列席人员提出意见。

“十一五”时期的工作回顾

2006年至2010年，市政府在市委的正确领导下，在市人大及其常委会和市政协的监督支持下，认真贯彻落实党中央、国务院和省委、省政府的决策部署，团结带领全市人民认真学习实践科学发展观，战胜了特大自然灾害，有效应对国际金融危机冲击，抢抓机遇力口快发展，圆满实现了2010年经济社会发展主要预期目标，较好地完成了市第十一届人大三次会议通过的“十一五”规划任务，为推动经济社会跨越发展打下了良好基础。

一、过去五年，是经济持续平稳较快增长、人民生活水平不断提高的五年 据统计，2010年全市实现生产总值683.1亿元，比上年增长12.5％，连续5年经济增长率高于全国、全省；比2005年增加346亿元，五年年均增长12.7％，连跨400亿元、500亿元、600亿元

三个台阶。2010年全市人均生产总值突破2万元、达2．3万元，增长12.1％，年均增长12.2％。来源于韶关的财政总收入143.3亿元，地方财政一般预算收入47.8亿元，分别增长32.4％和17.5％，年均分别增长18.2％和19.1％。年末金融机构本外币各项存款余额907.7亿元，贷款余额376.1乙元，年均分别增长14.2％和17.3％。县域生产总值和地方财政一般预算收入年均分别增长15％和24.2％，呈现加快发展的新局面。

2010年城镇居民人均可支配收入14260元，农村居民人均纯收入6317元，分别增长10.1％和18.3％，五年年均增长10.6％和11.1％。城乡居民储蓄存款余额562.1亿元，增长14.8％，年均增长15.1％。城镇居民恩格尔系数下降到39％。韶关市区居民消费价格指数涨幅为2.9％，低于全国、全省上涨水平。农村居民人均住房面积23．06平方米，市区城镇居民人均住房建筑面积33．8平方米，分别比2005年末增加4.46平方米和6.44千方米。年末市

区每百户居民拥有小汽车12辆，比2005年增长11倍；移动电话用户211.4万户、家庭宽带用户21.2万户，分别比2005年增长5 倍和4.5倍。

二、过去五年，是投资规模快速扩大、发展后劲明显增强的五年

2010年完成全社会固定资产投资433.7亿元，增长21.7％，五年年均增长25％。五年累计完成投资1460亿元，比“十五”时期增加923亿元。全力推进“十大工程”建设，五年累计完成投资812亿元，建成项目93个，在建项目50个。“十一五”期间，建成高

速铁路118公里，高速公路125.3公里，一级公路85.3公里，发电装机容量85.5万千瓦，防洪堤232．7公里，污水处理能力26万吨／日，垃圾处理能力1200吨／日，旅游接待床位8000多张；钢铁、铅锌年生产能力分别达到600万吨和45万吨。

交通枢纽、能源、水利基础设施和现代产业项目建设取得重大成果。交通重点项目五年累计完成投资240多亿元。武广高铁(韶关段)、韶赣高速公路(粤境段)建成通车，京港澳高速公路(韶关南段)扩建全面完成，赣韶铁路(粤境段)、广乐高速公路(韶关段)建设加快推进，新建、改建国省道210.5公里，高等级路面比例大幅提升。500千伏坪B线路工程等一批电网建设与改造工程完成，坪B电厂三期2x30万千瓦机组投产。湾头水利枢纽工程竣工运行，大中型水库除险加固工程基本完成，乐昌峡水利枢纽工程加快建设，全市防灾减灾能力明显提高。东阳光光箔系列产品扩建、丹冶技改一期、乳源氯碱化工基地一期和旭日玩具城二期、曲江至卓飞高线路板厂二期等项目建成投产，韶钢节能减排项目全面推进， 比亚迪汽车零部件、东阳光生物制药等项目建设进展顺利。韶关发电厂“上大压小”2X60万千瓦机组项目获得核准，韶关核电等项目前期工作扎实推进。

三、过去五年，是经济发展方式加快转变、生态文明建设取得显著成效的五年

2010年末全市市场主体总数达9.87万户， 比2005年末增加3.1万户。全市有26个项目列入省现代产业500强，居全省第6位。三次产业结构由 2005年的16.4：42.6：41调整为13.7：42.6：43.7，先进制造业增加值占工业增加值的比重提高到31．7％，五年高新技术产业产值年均增长20％。2010年全市完成工业增加值252．4亿元，五年年均增长12.9％，钢铁、有色金属、烟草、机械、电力、玩具、制药等七大支柱工业不断发展壮大，实现增加值占规模以上工业的77％。2010年服务业增加值298.4亿元，年均增长14.9％。接待旅游者人数1582万人次，旅游收入106.8亿元，分别增长29％和49.7％，比2005年增长3.1倍和5倍，旅游收入年均增长速度居全省第一位。我市被评为中国旅游竞争力百强城市、2008--2009年度中国最具人气旅游城市、中国精品休闲度假旅游城市和首届广东最受欢迎自驾游目的地，曹溪温泉度假村、广东大峡谷、云门寺佛教文化生态保护区成为国家 4A 级景区。2010年全市社会消费品零售总额329.8亿元、增长18.8％，比2005年增长1.3倍；商品房销售额比2005年增长4.3倍。民营经济较快发展。2010年民营经济完成增加值322.1亿元，年均增长12.3％；年末全市个体工商户户数比2005年增长51.6％，私营企业户数增长93.4％、注册资本增长1.68倍。

我市在全省山区市中率先通过国家科技进步考核，率先创建科技企业创业园(孵化器)。“十一五”期间，全市专利申请量、授权量分别比“十五”期间增长2倍和3倍，连续5年居全省山区市首位。成功创建韶关(省级)高新技术产业开发区。创建省部产学研创新联盟2个， 成立 博士后科研工作站2个，成立工程技术研究开发中心30家。实施科技项目600多项，获国家科技进步奖1项、省科学技术奖22项。企业科技成果转化率达90％以上。创

中国驰名商标1件、中国名牌产品1个，均实现零的突破。至2010年底，全市拥有省著名商标22件，注册商标总数4569件，分别比2005年增加21件和2990件。

我市成为全国首批生态文明建设试点地区。2010年末森林覆盖率达71.5％，森林资源数量和质量均居全省首位。五年累计治理水土流失面积30平方公里，建成污水处理厂11个。2010年末县以上城镇生活污水处理率达69.9％。提前一年完成集体林权制度改革，极大地调动了林农发展林业生产、维护林业生态的积极性。2010年全市地表水功能区达标率为92.3％，集中式饮用水水源地水质达标率达100％，空气质量达到国家二级标准。始兴、

乳源被省委、省政府授予南岭山地森林生态及生物多样性功能区生态发展试点县称号。乳源南水湖获批建设国家湿地公园。五年完成造林作业面积189.5万亩，关停落后钢铁产能83.3万吨、小火电73.2万千瓦，淘汰落后水泥产能195万吨，建成火电脱硫工程142.5万千瓦。2010年全市万元生产总值能耗下降到1.71吨标煤，五年累计下降20.1％；二氧化硫排放量和化学需氧量累计分别下降24．1％和9．4％，全面完成“十一五”节能减排目标任务。

四、过去五年，是“双转移”工作打开新局面、对外开放取得新进展的五年

2010年全市承接产业转移项目440个，合同投资304.4亿元、增长137％，到位资金101.7亿元、增长27.5％。2008—2010年累计承接产业转移项目1206个，合同投资540.1亿元，到位资金250.8亿元。2010年全市省级产业转移工业园规模以上工业增加值增长37.5％。东莞(韶关)产业园、东莞大岭山(南雄)产业园成功竞得省专业性产业园建设扶持资金，成为区域经济重要增长极。东莞大岭山(南雄)产业园、仁化有色金属循环经济产业基地成为全省首批省市共建循环经济产业基地。省市共建粤北现代技工教育基地取得实质性进展。2010年完成农村劳动力技能培训2.84万人、转移就业9.5万人，五年累计分别达12.2万人和39.9

万人。有3805名农民工积分入户城镇。在省2009年度农村劳动力技能培训转移就业和产业转移目标责任考核中，我市分别居全省第一名和东西北地区首位(全省第三名)，连续两年在全省“双转移”考核中荣获“双优”。

2010年全市实际利用外资2.12亿美元、增长12.1％，五年累计8.5亿美元、年均增长14.9％；进出口总额15.7亿美元、增长31．6％，五年累计58．9亿美元、年均增长11．3％；出口总额6．6亿美元、增长13．9％，五年累计27．8亿美元、年均增长14．2％。在

全省山区市率先开通铁海联运班列。2010年口岸通关货运量556万吨，五年累计1953万吨、年均增长17．2％。我市与四川宜宾市、西藏林芝地区结为旅游合作友好城市，与澳大利亚宝活市、韩国荣州市结为国际友好城市。

五、过去五年，是“三农”工作扎实推进、扶贫攻坚取得显著进展的五年

2010年农业增加值93．9亿元、增长5．6％，五年年均增长4．4％，高于同期全省平均水平。五年累计除险加固水库409宗，完成农村机电排灌工程189宗，新增节水灌溉面积8．4万亩，维修渠道1198．6公里；整治农田面积36万亩，开发耕地20万亩，连续9年实现耕地占补平衡。农业结构不断优化，粮食连续5年增产，2010年达91．5万吨。创建粤台(韶关)农业合作试验区，推进省市共建粤北农业现代化示范区。优质水稻、优质蔬菜、优质畜禽、特色精品农业等四大产业基地建设成效显著，优质稻、蔬菜、优质畜禽、烟叶、优质鱼、特色水果、竹子等七大主导产业发展壮大，园艺花卉、油茶等产业快速发展。2010年市级以上农业龙头企业达55家，农民专业合作社达497家，分别比2005年增加20家和480家；全市拥有国家地理标志保护产品12个、居全省第一(并列)，广东省农业名牌产品10个，无公害农产品、绿色食品、有机食品210个，产地农产品抽检合格率达95％以上。

五年累计完成农村公路硬化4500多公里，农村客运通村率85％；解决35万人饮水安全问题；建成农家书屋694间，农民体育健身工程近1200个；帮助5万户农民改建住房、新建户用沼气池12万个，2010年末全市有63．4％的农户住上楼房或红砖瓦房，17．4万农户用上沼气。乡村“清洁美”工程和“村庄整治”工程深入开展。在全省率先开展扶贫开发“双到”小额贷款贴息工作。2010年落实"7／到”工作帮扶资金3．43亿元，实施扶贫项目1071个，贫困村、贫困户的生产生活条件明显改善；355个贫困村的贫困户人均增收688．3元，78383人实现脱贫， 占贫困人口的54．4％。

六、过去五年，是城市建设加快推进、城市功能明显增强的五年

2010年，韶关市区建成区面积81．83平方公里，供水、供气、排污、公交等市政基础设施明显改善。五年累计新建、改建市政道路27条共58．2公里，更新公交车223辆，新增公交线路12条，新增绿地面积569．1公顷。2010年末绿地率达43．1％，人均公共

绿地面积11．75平方米。五年来，芙蓉新城武广高铁韶关站及站前广场、京港澳高速公路韶关互通、韶关大道、国道323线市区过境段、宝盖路、黄金村大桥、帽峰大桥等重大基础设施建成使用。韶州公园、芙蓉山国家矿山公园、韶阳楼、城市规划展示厅、韶州公园绿道等建成开放，林桥坑整治一期工程竣工，芙蓉大道、滨江路、东环路等项目开工建设，东堤路百年东街等“三旧”改造项目有序推进。我市荣获全国双拥模范城、国家卫生城市、国家园林城市、中国金融生态城市等称号，全面启动全国文明城市创建工作。

七、过去五年，是保障和改善民生成效显著、社会文明进步的五年

五年来，韶关人民战胜了超百年一遇的特大洪灾、历史罕见的雨雪冰冻灾害等多次严重自然灾害。全市累计投入救灾专项资金5亿多元，救济灾民289．7万人次，帮助灾民建房29144套。去年翁源等地((5· 6” 洪灾全倒户已全部搬进新居。五年累计城镇新增就业26．3万人、城镇失业人员再就业17．7万人，去年城镇登记失业率2．99％。建立被征地农民社会保险、城镇老年居民养老保险和城镇居民基本医疗保险等制度。新型农村养老保险试点范

围扩大，新型农村合作医疗基本实现全覆盖。提高了城乡低保、企业退休人员基本养老和优抚对象等人员的生活补助标准。五年累计投入低保资金4．3亿元、惠及52．4万人次。医疗救助40．8万人次，救助各类流浪乞讨人员25348人次，办结法律援助案件4318件。建成地质灾害气象预警预报系统、地震监测网络和4个市级地震应急避难场所。五年建成保障性住房2947套，基本解决市区和县城低收入家庭住房困难问题。

2008年在全省东西北地区率先普及高中阶段教育。2009年启动创建广东省教育强市工作。五年累计有20个镇(街)通过省教育强镇督导验收，全市新增校园面积72．3万平方米，建成义务教育规范化学校217所。学前教育、特殊教育和民族教育协调发展，高等教育规模逐步扩大。教育质量和办学水平居全省东西北地区前列。城乡基层医疗卫生服务体系进一步完善，重大疾病得到有效控制，连续五年全市没有发生重大甲、乙类传染病暴发疫情和重大食物中毒事件。医药卫生体制改革稳步推进。全市以街道为单位的社区卫生服务中心实现全覆盖，村卫生站覆盖率达99．4％。每千人口拥有医院床位数居全省前列。人口与计划生育

工作水平不断提高，成为全国首批人口计生综合改革示范市，人口自然增长率控制在7％o以内，低生育水平保持稳定。启动粤北区域文化中心建设，文化事业加快发展。大批文艺精品获国家级或省级奖励，《拜盘王》、《瑶族刺绣》等16个项目入选国家级或省级非物质文化遗产名录。抗战时期中共广东省委暨粤北省委机关旧址修复保护项目和北伐战争纪念馆建成开放。仁化县石塘村荣获“中国历史文化名村”称号。我市通过了广东省二类城市语言

文字工作评估。市区有线数字电视整体转换基本完成。成功承办广东省第四届少数民族传统体育运动会，我市代表团夺得团体总分第一。全民健身运动、竞技体育、体育产业协调发展。乳源成功承办第十一届中国瑶族盘王节。

社会主义核心价值体系建设扎实推进，社会和谐文明稳定局面进一步巩固。全市超过70％的社区成功创建“六好”平安和谐社区。全面完成省、市《妇女儿童发展规划(2001—2010年)》目标任务，妇女儿童教育和健康水平明显提高，保障妇女儿童生存发展的社会环境不断优化。全面完成市、县两级人民来访接待厅和县(市、区)、镇(街)、村(居)三级综治信访维稳中心建设，群众上访和矛盾纠纷逐年减少。应急管理机制进一步完善，突发事件处置能力提高。社会治安状况进一步好转，刑事案件同比前五年下降23．3％。产品质量、食品药品监管和安全生产工作加强。较好地完成了第二次全国经济普查和第六次全国人口普查在韶关范围内的普查任务。国防建设、人民防空工作取得新成绩，完成了地面应急指挥中心和机动指挥所建设，提高了战时防空能力。民族宗教、对台、外事侨务、港澳、统计、审计、物价、供销、气象、水文、新闻出版、广播电视、无线电管理、档案、史志、残疾人、老龄、移民、红十字会等各项工作都有新的进展。

八、过去五年，是民主法制建设不断加强、人民群众对政府满意度继续上升的五年

市政府自觉接受市人大及其常委会的监督，落实人大及其常委会各项决议决定，坚持向人大及其常委会报告工作，主动接受市政协的民主监督，向市政协通报工作情况，支持人大代表和政协委员履行职责。五年共组织实施人大代表议案7件。共办复人大代表建议475件、政协建议案21件、政协委员提案582件和委员来信330件，办复率和办结率均为100％，人大代表、政协委员普遍表示满意或基本满意。大力支持工会、共青团、妇联等人民团体开展工作，力口强与各民主党派、工商联和无党派人士的联系。深入推进政府信息和政务、事务、厂务、村(居)务公开。顺利完成市、县两级新一轮政府机构改革，启动南雄省直管县财政改革试点和乳源深化县级行政管理体制改革试点， 富县强镇和简政强镇事权改革扎实推进。开展综合行政执法体制改革试点。推进依法行政，加强执法监察，不断规范行政行为，促进依法治市。完成“五五”普法。五年共发布实施市政府规范性文件64个。行政审批事项由594项核减到159项，基本完成市行政审批电子监察系统二期项目建设。市、县两级行政服务中心管理规范，窗口服务群众满意率保持在99％以上。加大廉政监察和效能监察力度，专项整治“小金库”、商业贿赂、涉企涉农乱收费、公路“三乱”和工程建设领域突出问题，有效纠正损害群众利益的不正之风。大力推进机关作风建设，建立健全绩效考评机制。严肃查处了一批违纪违法案件。全面开展网络问政，办好市长信箱和“民声热线”，解决了大量关系群众切身利益的热点、难点问题。坚持为民办实事，五年共办理44件。去年承办的十件实事中，除林桥坑二期整治、市中心业余体校和粤北第三人民医院住院大楼因工程较大还在抓紧实施外，其余7件已完成或基本完成。

各位代表：上述成绩来之不易，这是全市人民团结奋斗、社会各界支持配合的结果。在此，我代表市人民政府，向全市人民，向驻韶人民解放军和武警官兵，向省直部门和驻韶中省单位，向各民主党派、工商联、无党派人士和各人民团体， 向所有关心、支持和参与韶关发展的各界朋友表示衷心的感谢!

在看到成绩的同时，我们也清醒认识到，前进中还面临不少困难和问题。主要有： 一是经济增长方式比较粗放，总量较小，加快发展、增强实力的任务十分紧迫；二是产业投资规模不大，扩大招商引资、增强发展后劲的任务十分迫切；三是中心城市带动能力不强，农业农村基础薄弱，统筹城乡发展、推进扶贫开发的任务仍然艰巨； 四是社会建设、管理和服务有待加强，改善民生、维护稳定等任务仍然艰巨；五是行政效率和服务水平还要进

一步提高，投资环境需要进一步优化，思想观念也要进一步解放更新。对此，我们一定要高度重视，并采取措施切实加以解决。

“十二五”时期的主要任务和2011年工作意见

“十二五”时期是我市走生态文明发展道路、实现经济社会跨越发展、建设幸福美好韶关的关键时期，也是工业化城市化加快、信息化市场化加快和经济社会结构调整加快的重要转型期。当前，虽然国际金融危机影响仍然存在，但世界经济将在调整中逐步恢复增长，经济全球化趋势不可逆转；我国将以科学发展为主题，加快转变经济发展方式，促进经济长期平稳较快发展和社会和谐稳定；我省深入实施珠三角《规划纲要》、《东西北地区经济社会发展规划纲要》和《关于促进粤北山区跨越发展的指导意见》，加快实施提升珠三角带动东西北战略，促进区域协调发展、绿色发展、和谐共享力度进一步加大；我市经过“十一五”时期的较快发展，交通区位优势进一步凸显，“双转移”和产业建设取得重要进展，良好的发展态势已经形成。同时，国际经济形势复杂，国内宏观经济不确定因素较多， 区域经济竞争激烈，我市正处在调整转型、爬坡越坎的关键阶段，面临不少困难和挑战。必须抢抓机遇，齐心协力，克服困难，真抓实干，奋力开创跨越发展新局面。

根据市委关于制定“十二五”规划的建议，市政府组织编制了《韶关市国民经济和社会发展第十二个五年规划纲要(草案)》，提出今后五年要顺应人民群众过上幸福美好生活新期待，以科学发展为主题，以加快转变经济发展方式为主线，围绕“探索一条生态文明发展路子，狠抓人力资源培训和配置、提高城市和产业集聚度这两个关键，突出大交通、大旅游、大产业三大重点，加快推进新型工业化、新型城市化、特色资源产业化和生态建设系统化”的基本工作思路举措，坚持加快发展、生态优先、城乡统筹、开放创新、改善民生的基本要求，加快建设粤北区域中心城市、广东先进制造业基地、优质农产品生产加工基地、全国生态旅游休闲重点地区、全国生态文明建设示范市，增强综合竞争力，努力实现经济社会跨越发展，建设幸福美好韶关。力争到2015年，全市生产总值达到1200亿元、年均增长12％以上，三次产业比例约为10：45：45，人均生产总值达到3．85万元、年均增长11％以上，粤北区域中心城市初步建成，现代产业体系基本形成，自主创新能力大幅提升，社会事业全面进步， 民主法制日益健全，人民生活水平进一步提高，社会更加和谐稳定；力争到2015年，生态文明建设成效明显，节能减排任务全面完成，全市森林覆盖率达到72％，城乡环境质量稳步提高，初步构建生态经济体系、生态文化体系、生态人居体系，基本建成全国生态文明建设示范市，资源节约型、环境友好型社会建设取得显著成效，韶关天更蓝、山更绿，水更清，可持续发展优势更强。

为实现以上目标，突出抓好三大重点建设：

一是建设大交通，打造名副其实的粤北区域中心城市，推进新型城市化。加快构建以“六高四铁两航两站”为骨架的立体交通网络，基本建成且家公路运输枢纽和一级铁路枢纽城市。加快芙蓉新城开发建设，引导人口集聚，促进城市“一心五组团”格局形成。稳步推进城乡基本公共服务均等化，着力保障和改善民生，提高人民群众幸福指数。力争到2015年，市区常住人口达到100万人左右、建成区面积100平方公里左右，成为集交通枢纽、产业集聚、物流商贸、旅游休闲、医疗服务、文化教育六大中心于一体的粤北区域中心城市，为建设粤北山区及粤湘赣相邻地区中心城市和韶关都市区打下坚实基础。

二是建设大产业，打造广东先进制造业基地、优质农产品生产加工基地和三省边界物流中心，加快新型工业化，构建现代产业体系。在大力改造提升传统优势产业的同时，狠抓招商引资，培育发展一批战略性新兴产业，加快建设韶关高新区、省级产业转移工业园、粤北现代农业示范园区、科技企业创业园等产业载体，力争早日建成广东清洁能源生产基地、世界锌都、钢铁生产基地、汽车及零部件生产基地、装备基础零部件先进制造业基地、新型干法水泥生产基地、现代中药和生物医药产业基地。同时，充分发挥自然条件和区位交通优势，大力发展现代农业和物流等生产性服务业。

三是建设大旅游，打造全国生态旅游休闲重点地区。着力引进战略投资者，培育一批旅游龙头企业，加快旅游特色资源产业化，推动旅游产业聚集发展，推进粤北旅游圈建设，打响“丹霞山世界自然遗产地”和“禅宗文化圣地”两大品牌，初步建成生态休闲、户外运动两大基地，构建大旅游发展格局，率先实现旅游业跨越发展。

今年是中国共产党建党九十周年、“十二五”规划的开局之年。根据市委十届九次全会精神，围绕“十二五”发展目标任务，2011年政府工作的总体要求是：以党的十七届五中全会和中央经济工作会议精神为指导，按照省委十届八次全会的部署，紧紧围绕加快转型升级、建设幸福广东这个核心，以科学发展为主题，以加快转变经济发展方式为主线，充分发掘我市特有优势，力争在产业转型升级、探索生态文明发展、壮大县域经济、建设区域中心城市和创新体制机制上取得新突破，着力保障和改善民生，全面发展社会事业，筑牢跨越发展的基础，为顺利实施“十二五’’规划、建设幸福美女子韶关开虫子局起好步，以优异成绩迎接建党九十周年。

经济社会发展主要预期目标是：生产总值增长12％，人均生产总值增长11％，全社会固定资产投资增长16％以上，社会消费晶零售总额增长15％，实际利用外资增长12％，外贸出口增长13％，地方财政一般预算收入增长8％(按可比口径计算增长12％)，城镇居民人均可支配收入增长8％，农村居民人均纯收入增长8．5％，居民消费价格指数涨幅控制在4％左右，服务业增加值占生产总值比重达44％，研究与发展经费支出占生产总值比重提高到1．6％，人口自然增长率控制在7．6％0以下，城镇登记失业率控制在3％以内，完成省下达的节能减排任务。

围绕实现以上目标，要突出抓好以下九个方面的工作：

一、强力推进招商引资，力争大规模承接产业转移

狠抓招商引资工作。优化招商环境，强化目标管理，完善奖惩机制，落实招商责任，形成招商合力，扩大招商成效，全年引进市外境内到位资金130亿元以上，实际利用外资2．3亿美元以上。突出招商引资重点，围绕加快发展先进制造业、现代农业和现代服务业开展产业招商，进一步加强促进中央和省属企业在韶投资工作，加强与珠三角的产业对接合作，重视吸引长三角民间资本。精心办好各类招商活动，积极参与省面向大型央企、国内民企100强和世界500强企业的三场重大招商活动，大力承接国内外产业转移。加大重点产业招商工作力度，积极开展产业链招商和特色产业集群式招商，旅游、物流、文化产业招商要有新突破，其他重点产业招商要有新进展。坚定不移地走质量招商之路，突出招大引强，努力在引进优质项目上取得新成绩。尤其要大力引进工业投资特别是战略性新兴产业投资项目，力争承接产业转移工作始终走在全省前列。

狠抓项目落地工作。加强分类指导，抓好衔接工作。深入推进行政审批制度改革，研究建立行政审批事项动态评估、管理和调整制度，推行网上审批模式。推进行政审批事项集中管理、规范审批程序，在重点项目建设和企业登记注册领域开展并联审批改革。进一步创新服务方式，加强统筹协调服务。围绕落实大项目，建立“一个项目一个责任领导、一支服务队伍”机制，千方百计加快实施。加强已签约项目的跟踪服务，加强已开工项目的配套服务，提高项目履约率、资金到位率和开工投产率。

大力加强园区建设。强化规划引导和政策扶持，做好征地拆迁工作，扩大产业转移工业园开发面积，完善配套建设和服务，提高产业承载力。抓好园区项目储备和土地储备，推动产业项目向园区聚集，促进产业聚集发展，尤其是促进韶赣高速公路沿线产业聚集。重点加强东莞(韶关)、东莞大岭山(南雄)产业园建设，创新园区管理和共建机制，做大机械装备制造、精细化工等主导产业，示范带动全市工业园区发展壮大。支持县域加快产业转移园区建设，打造精细化工、环保涂料、有色金属、制笔研发、氯碱化工、纺织、钟表等产业基地，培育发展特色产业集群，加速县域工业化进程。

加快外经贸发展。进一步转变外贸发展方式，培植壮大外贸经营主体，支持企业巩固拓展市场，优化外贸进出口商品结构，推进加工贸易转型升级。启动粤北国际物流中心项目建设，打造铁海联运品牌。完善口岸功能布局，推进口岸大通关建设。加快进口我市急需的关键设备和原材料，推动经济结构优化升级。深入实施“走出去”战略，提升对外经济合作水平。

二、努力扩大投资规模，加快重点项目建设

深入实施重点项目带动战略，着力扩大投资规模，优化投资结构。加大基础设施、现代产业、民生保障和生态环保等重点领域投资力度，精心策划一批战略性、基础性项目，扩大有效投资。切实把战略性新兴产业项目投资建设摆在突出位置，确保工业投资不低于全社会固定资产投资增幅，加快构建基础设施体系和新型产业体系。发挥重点项目投资的乘数效应，抓好60个重点项目建设，努力确保完成计划投资217．6亿元，力争全社会固定资产投资超过500亿元。抢抓省委、省政府“掀起粤东西北交通建设大会战”的机遇，加快国家交通枢纽和广东清洁能源基地建设，抓好广乐高速公路(韶关段)、赣韶铁路(粤境段)等续建项目和韶关发电厂“上大压小”项目建设，推进北江航道整治和港口、客运站场、公交枢纽站建设，力争大广高速(新丰段)、昆汕高速(翁源段)、粤湘高速(韶关段)、华电(南雄)热电冷联供等项目早日开工建设。加强韶关核电、韶关机场等重大项目前期工作。汛期前基本完成城乡水利防灾减灾及病险水库除险加固工程建设，乐昌峡水利枢纽工程要形成防洪能力。加强项目开发、争取、储备和要素保障，破解土地征用、环评和报批难题。完善重点项目推进机制，强化项目督查和责任考评，努力确保项目顺利实施。

加强财税金融工作。加强税收征管和纳税服务，大力扶持培育税源型企业，确保财税收入稳步增长。稳步推进非税收入管理改革。优化财政支出结构，增强预算执行力，保障重点支出需要。切实改善公共资产和公共资源的管理工作，提高管理水平和效益。加强财务管理，严肃财经纪律。积极争取中央和省的资金，规范政府投资项目管理，发挥政府投入的带动效应，着力扩大民间投资，鼓励民间资本进入基础产业和战略性新兴产业。进一步完善银政企联席会议制度，促进银企有效对接，保持贷款较快增长。完善莞韶公司、城投公司等多元投融资平台，发挥政策性担保公司作用，扩大融资规模。力争国有控股企业、中小企业改制上市融资有实质性突破。进一步优化金融生态环境，吸引金融机构进驻，加快金融创新发展，全面提升金融服务水平。有效增加财政投入和信贷投入，多渠道扩大有效投入。

三、全力加快新型工业化，提高自主创新能力

全力以赴加快工业发展，加速新型工业化进程。继续落实市领导联系重点企业制度，做好工业经济运行监测和煤电油运等生产要素保障工作，开展“工业绿灯”行动，加强对新投产企业和在建工业项目的服务，全力稳定和扩大企业产能。加大国有企业改革工作力度。加强列入省现代产业500强项目建设，培育壮大先进制造业，着力优化工业结构。大力实施产业集聚工程，发挥大型企业的龙头作用，促进中小企业协作配套，延伸产业链，加快省市共建装备基础零部件(韶关)先进制造业基地和省级产业集群升级示范区建设，着力培育百亿元企业、百亿元产业，加快钢铁、铅锌、铝箔、汽车配件、装备制造、玩具、精细化工等产业

集群化，做大做强优势特色产业。

增强自主创新能力，加快建设创新型韶关。坚持制造与创新并重，发挥企业科技创新主体作用，推进信息化与工业化融合，加强企业技改创新，运用高新技术促进钢铁、有色金属、烟草、机械、电力、玩具、制药等传统优势产业加快转型升级。加大技术改造和技术创新投入，支持企业技术进步和科研机构发展，完善区域创新体系。大力推进韶关高新区建设，加快高新技术产业发展。加强产学研合作，充分发挥省部产学研创新联盟的技术优势，支撑主导产业发展。加快建设华南理工大学韶关技术研究院和中科院广州化学所技术创新与育成中心，推进企业科技特派员工作。加强工程技术开发中心、企业技术中心、行业公共创新平台等建设，启动科技企业创业园三期工程，吸引先进技术项目入园转化和产业化。启动实施全市液压机械装备产业技术路线图。依托优势产业、特色产业，联合高校、研究院所建立公共服务平台。大力实施新兴产业倍增工程，加强战略性新兴产业项目引进和建设，加快建设乳源东阳光生物制药基地、南雄精细化工基地、莞韶生物医药科技园、曲江LED产业基地、仁化有色金属循环经济产业基地等新兴产业基地，培育生物医药、新能源、节能环保、新材料等战略性新兴产业。深入开展质量强市活动，大力实施知识产权战略、技术标准战略和名牌带动战略。抓好省级铅锌及钢材产品、粮油及制品、精细化工产品检验站、粤北玩具检测中心省级重点实验室建设， 申报国家铅锌质检中心，完善公共检验检测技术服务平台。

着力扶持民营经济加快发展。认真落实国家和省、市促进中小企业、民营经济发展的政策。加强引导和服务，完善社会化服务体系，进一步优化民营经济发展环境。大力吸引民间资本进入第三产业。减轻企业负担，落实财政扶持资金投入。完善中小企业金融指导员制度，加强中小企业信用体系建设，健全中小企业融资服务体系，引导金融机构加大信贷支持力度，缓解中小企业融资难题。支持民营企业技改创新，加快转型升级步伐。大力实施民营企业上市梯度培育工程和企业家素质提升工程，深入实施中小企业成长工程，加快推进省级中小企业创业基地、民生创业园等创业载体的配套设施建设，推进民营工业聚集发展。力争民营经济增加值占生产总值的比重达到50％。

四、大力发展现代农业，深入推进扶贫开发

加快农业现代化。全面落实强农惠农政策，加大对农业农村的投入，稳定粮食生产，大力发展现代农业，促进农业增效、农民增收。加快改造提升蔬菜、畜禽、烟叶、水产、竹子、油茶等优势特色产业，积极培育休闲观光农业，加快发展高效生态农业，提高产业化、集约化、标准化和生态化水平。力口快粤北现代农业示范园区、粤台(韶关)农业合作试验区和华南大宗农产品物流交易中心建设。积极引进和扶持农业龙头企业，规范发展农民专业合作组织，培育发展种养大户。加强种养基地建设，健全农产品质量安全保障、质量溯源管理和标准体系，大力发展无公害、绿色、有机农产品和地理标志保护产品，培育特色农业品牌。规范发展各类林场，大力发展园艺花卉、珍贵树种和中药材种植业，提高林分质量，推进林相改造，加快现代林业发展。推进土地开发整理和中低产田改造、现代标准农田建设等农业综合开发，加大灌渠改造力度，加快民生水利工程建设，改善农田水利基础设施。建立与完善产业技术服务平台，推动专业镇转型升级。加强基层农技推广和动植物疫病防控及病虫害防治等工作，加快农业机械化，全面提高现代农业物质技术装备水平。加强气象灾害预

警应急工程建设，提高防灾减灾能力。

全力打好扶贫攻坚战。推进山水田林路综合治理，加强农村基础设施建设和社会救助等基本公共服务，进一步。改善贫困村的生产生活条件。认真落实“双到”政策，突出抓好产业扶贫和技能培训，提高贫困人口自我发展能力，确保今年全市80％以上的被帮扶人口年人均纯收入2500元以上，被帮扶村的落后面貌有明显改观。加快革命老区、少数民族地区、边远山区和水库移民区发展。继续落实十项农民福祉工程，进一步改善农村生产生活环境，加快社会主义新农村建设。加强村庄整治，创建20个市级和100个县级整治示范村，建设一批具有粤北山区特色的宜居村庄。基本实现全市镇级有站、符合通客运班车条件的行政村100％通客车和100％有候车亭的目标。完成80宗农村饮水安全工程和1万户以上农村住房改造。

深化农村综合改革，激活农村发展活力。推进城市基础设施和公共服务向农村延伸，推动城乡基本公共服务一体化，促进县域经济加快发展。加强镇、村规划编制，推进城乡规划全覆盖。坚持和完善农村基本经营制度，依法推进农村土地流转。深化征地制度改革。推进农村集体土地所有权确认工作。依法加强农村宅基地管理。积极推进政策性农业保险。拓宽农民增收渠道，努力增加农民生产经营性、政策转移性、工资性、财产性等方面的收入，促进农民收入持续增长。

五、全面推进旅游“二次创业”，促进服务业加快发展

大力推动旅游业提质升级。充分利用丹霞山成功申遗效应工发挥交通区位优势，整合全市旅游资源，完善城市旅游功能，加快省市共建旅游信息化示范区建设，大力推动旅游业从观光旅游向观光、休闲、文化、健身、购物等复合型旅游发展方向转变，加快旅游“二次创业”，推进旅游产业化，发展大旅游产业。千方百计引进若干战略投资者，进一步加快以大丹霞、大南华、大南岭、大珠玑和世界过山瑶祖居地为重点的知名生态休闲旅游目的地建设。承办好“2011广东国际旅游文化节”。推进丹霞山创国家 5A 级旅游景区、全国文明景区工作。继续推进星级饭店和A级景区创建，建设全市旅游咨询服务网络，完善旅游公共服务体系。力争创建国家 4A 级旅游景区3家，新增五星级标准酒店3家。抓好旅游宣传促销，大力开拓新的客源市场，努力发展入境游。加强旅游人才建设，创新旅游管理体制机制，规范壮大各类旅游企业主体。鼓励支持旅游商品开发、设计、生产和销售，大力发展旅游购物市场。

加快物流业等现代服务业发展。完善综合交通、物流信息、现代流通三大物流网络平台，加强商贸物流、商务会展、科技服务载体建设，加快建设一批物流园区。重点推进曲江城北、韶关雪印、乐昌粤湘、始兴交通物流中心、仁化周田大宗农产品等物流园区或项目建设。积极培育引进龙头企业，支持一批物流企业做强做大。发展新兴专业市场，完善网点布局和．业态结构，加快建设粤北商贸物流大走廊和三省边界物流中心。同时，推进服务业与制造业有机融合、互动发展，做大做强生产性服务业。

促进消费结构升级。深入实施扩大内需战略，促进城乡消费繁荣。积极健全市场体系和社会信用体系，继续严厉打击制售假冒伪劣产品行为，规范市场秩序，优化消费环境。深入实施“农村消费升级行动计划”、“万村千乡市场工程”和“新网工程”，推进“农超对接”，推动现代流通方式由城市向农村延伸。加快多层次的住房保障体系建设，规范房地产开发秩序，促进住房消费健康发展。继续做好家电下乡、以旧换新工作。巩固汽车、信息、商贸、餐饮消费，扩大文化旅游休闲消费，加快发展电子商务、物流配送、网上交易等新型消费业态，推进信贷消费。认真落实“菜篮子”市长负责制，完善重要商品储备制度和主要农产品临时收储制度，加强价格调控监管，打击各种价格违法行为，着力保障市场有效供给和物价稳定。

六、加强宜居城乡建设，开创生态文明建设新局面

加快芙蓉新城建设。加快完善和实施芙蓉新城规划，做好行政第二组团规划定点及控规编制，抓好金融机构、大型企业总部进驻新城规划选址工作。完成新城的征地收尾工作，全面启动安置区建设，保障新城重点项目用地，加快基础设施和公共服务设施建设，启动芙蓉隧道等工程建设。积极开展融资工作，吸引社会资金投入新城开发建设。

改造提升城市功能。编制实施小岛片区旧城控制性详细规划，科学谋划和推进小岛片区改造。稳妥推进“三旧”改造，开展示范点项目建设。继续推进东环路建设和原曲仁矿棚户区改造，完成百年东街改造、仙桥古渡和客家源流项目。加强市政基础设施和绿道网建设，优先发展城市公交，努力解决堵车塞车问题。全面提升城市信息化管理水平，优化城市环境，有效增加市区公共休闲空间。推进市中心区通往曲江区道路建设。支持县(市)城区和中心镇加快建设，完善基本公共服务，着力打造一批宜居名镇名村，加快城镇化进程。

加强生态建设和环境保护，加快创建全国生态文明建设示范市。高度重视林地林木资源保护管理，抓好“封山育林”工程和北江水源涵养林、珠江防护林建设，加强生态公益林体系、生态修复、 自然保护区、湿地保护和水土保持、石漠化治理、地质灾害防治等生态工程建设。推进始兴、乳源南岭山地森林生态及生物多样性功能区生态发展试点县工程，加，陕生态产业化和产业生态化。建立长效机制，深入开展乡村“清洁美”工程。建设200个“万村绿”工程示范点。实施节能减排重点工程，力口快产业园区和重点镇污水处理设施建设。大力倡导低碳生活方式和消费模式。做好电动汽车示范应用工作。加强清洁生产、节能降耗管理、淘汰落后产能和废物综合利用工作，确保全面完成节能减排任务。制定并严格实施土地供应计划，加强节约集约用地，积极推进城乡建设用地增减挂钩试点工作。严厉打击各类土地违法行为和非法采矿行为，强化土地执法问责。抓好地质灾害预测预报和防治，健全

环境污染事故预防和处置制度，坚决防止重大环境污染事件发生。

七、优化人力资源配置，着力保障和改善民生

加强农村劳动力技能培训转移就业示范基地建设，开展农村劳动力转移就业示范县创建活动。落实省市共建粤北现代技工教育基地建设项目，进一步提升劳动力技能培训转移就业工作质量。加强区域劳务合作，加大劳务输出力度，大力发展劳务经济。完善城乡公共就业服务体系，构建城乡一体化的人力资源市场。积极落实各项就业补贴、小额担保贷款和创业促就业等政策。加大就业援助力度，多渠道开发就业岗位，着力做好高校毕业生、农村转移劳动力、城镇就业困难人员和退役军人的就业工作。抓好省级创业孵化基地和浈江区、仁化县创业型城市创建工作，健全创业服务体系，提高劳动者创业能力。力争今年城镇新增就业5万人，就业困难对象实现再就业3500人。积极推行企业工资集体协商制度。深化人事制度改革，进一步加强人才和智力的引进工作，加快引进创新科研团队和科技领军人物。

进一步完善社会保障体系，扩大城乡基本养老、基本医疗、失业、工伤和生育等社会保险覆盖面。落实城镇老年居民养老保险新政策，完善被征地农民养老保险试行办法。逐步提高全民医保待遇水平。完善失业保险、工伤保险制度。扩大新农保试点范围，确保到2012年实现新农保全覆盖目标。推进农村低保与农村脱贫工作同步开展，逐步将人均年收入1500元以下的困难家庭纳入低保。健全完善优抚安置保障体系，实现优抚对象参保全覆盖。继续抓好保障性安居工程建设，不断改善城镇低收入困难群众的住房条件。探索对低保边缘家庭提供临时性应急性救助办法，加强社会救助工作。重视发展残疾人事业，加强残疾人社会保障和服务体系建设。规范捐赠资金管理，大力发展慈善事业。

八、创新社会服务管理，推进粤北区域文化中心建设

坚持教育优先，加快教育改革发展，推进教育强市建设。加大教育投入，实施义务教育规范化学校建设工程和学校卫生安全工程，推进义务教育均衡发展。巩固提高高中阶段教育成果。推进粤北区域职(技)教中心建设。实施学前教育三年行动计划，鼓励多元投资，加快学前教育发展，提高发展质量。加强特殊教育和民族教育，大力发展民办教育，支持高等院校提升办学水平，促进各类教育协调发展。全面实施素质教育。完善教师培养培训体系，建设高素质教师队伍。深化医药卫生体制改革，确保完成三年医改任务，初步建立国家基本药物制度，稳妥推进公立医院改革试点，进一步完善新农合管理，提高保障水平。加强乡镇卫生院和社区卫生服务机构建设，完善城乡基层医疗卫生服务体系，继续实施以建立城乡居民健康档案为切入点的医疗卫生信息化工程，大力落实基本公共卫生服务均等化项目，推进基本公共卫生服务逐步均等化。加强疾病预防控制、妇幼保健、卫生监督等工作，加强艾滋病等重大传染病防控，提高食品安全保障水平。实施“名院、名科、名医”和中医药强市战略，大力推进爱国卫生运动，积极推进省市共建粤北区域医疗服务中心工作。推进全国人口计生综合改革示范市建设工作，提升人口计生服务水平，促进人口均衡发展。做好《妇女儿童发展规划(2001—2010年)》终期评估验收工作，编制妇女儿童发展新规划。完善城乡体育基础设施，大力开展全民健身运动，协调发展群众体育、竞技体育和学校体育。加强国防教育、国防动员、民兵预备役和优抚安置工作，争创第九届全国双拥模范城。

加快建设粤北区域文化中心。加强精神文明建设，深入推进群众性精神文明创建活动，争创全国文明城市工作先进市。加强公民思想道德建设，深入开展爱国主义教育，倡导感恩奉献精神。深化文化体制改革，推进报纸、广播电视资源整合，组建市传媒集团；完成全市新华书店重组、县级电影公司改制和市县两级文艺院团改革任务，完成县、区广播电视网络改革重组工作，积极推进“三网融合”试点。推进重点文化惠民工程，加快市、县两级图书馆、文化馆、博物馆和乡镇(街道)综合文化站、行政村(社区)文化室等公共文化基础设施建设，完善公共文化服务体系。启动创建国家历史文化名城工作，加大对外文化交流力度，提高韶关文化的影响力。加强文化遗产保护和利用。鼓励支持创作文艺精品。大力发展特色文化产业，加快培育文化创意产业集群，推进市生态文化创意园、大南华文化创意产业园等项目建设。扶持文化企业和文化产业加快发展，促进文化行业健康发展。

深化社会管理体制改革。创新社会管理方式，推动管理重心下移，构建党委领导、政府负责、社会协调、公众参与的新型社会管理体制。深入推进城乡基层民主建设，完善乡镇治理机制和城乡群众自治机制。推进社区居委会规范化和社区服务专业化，规范社区物业管理。开展深化城市基层管理体制改革试点。建立政府购买服务机制。深化户籍制度改革，完善流动人口服务管理居住证“一证通”制度，加快推进农民工积分制入户城镇、子女积分入学工作。创新虚拟社会管理，规范发展社会组织。进一步加强安全生产监管和食品药品监管，完善应急管理体制，提高安全保障水平。用群众工作统揽信访工作全局，畅通民意表达渠道，积极排查调处化解矛盾纠纷，加大调解工作力度，提高基层综治信访维稳中心工作水平。加强社会治安综合治理，加大群防群治力度，整治社会治安重点地区和突出治安问题，健全社会治安防控体系，加大打击刑事犯罪力度，提高群众安全感，促进社会和谐稳定。继续做好民族宗教、外事侨务、新闻出版、对台、港澳、统计、人防、供销、水文、防震减灾、无线电管理、档案、史志、红十字会、老龄等各项工作。

九、着力提升政府效能，打造一流政务环境

坚持向市人大及其常委会报告工作，向市政协通报工作情况，自觉接受人大及其常委会、人民政协和社会各界的监督。认真听取各民主党派、工商联、无党派人士、人民团体和人民群众的意见建议。努力办好人大议案、人大代表建议、政协建议案和政协委员提案。推进政务公开和政府信息公开，创新公开方式，保障人民群众的知情权、参与权、表达权和监督权。深入调查研究，坚持并健全重大决策公众参与、专家论证、风险评估、合法性审查和集体讨论决定制度，加强政府层级监督，接受人民群众和新闻舆论监督，提高民主、科学、依法决策水平。加快创建法治城市和法治县(市、区)，推进法治政府建设，全面提高各级政府依法行政能力。强化行政问责，严肃追究失职、渎职、不作为和乱作为的责任。切实加强督查督办，着力确保令行禁止，提高政府执行力和公信力。稳步推进事业单位分类改革。深化县级行政管理体制改革，在乳源深化大部门制改革。继续推进富县强镇和简政强镇事权改革，增强基层活力。启动“六五”普法教育。进一步办好政风行风热线、市长信箱和“民声热线”，完善网络问政，着力解决人民群众反映强烈的突出问题。

全面正确履行政府职能，更加重视公共服务和社会管理，增强基本公共服务能力。精简会议和文件，严格控制各类评比和庆典活动。加强行政服务中心建设，进一步推动“窗口”单位服务向规范有序、公开透明、便民高效转变。加大审计监督力度，强化经济责任审计和绩效审计。加强行政监察工作，继续开展专项治理，坚决纠正损害群众利益的不正之风。加强政府廉政建设，推进反腐倡廉制度建设，加大从源头上预防和治理腐败的力度，强化对权力运行的制约和监督，严厉查处和惩治腐败。加强公务员培训，深入开展创先争优活动和廉洁从政教育，提高公务员综合素质，努力建设政治坚定、业务精湛、作风过硬、人民满意的公务员队伍。

今年还要办好八件关系民生的实事： 1、抓好农村电网升级改造；2、完成通自然村公路硬化400公里；3、实施韶南大道改造工程：4、实施天子岭廉租住房二期工程：5、健全完善我市四级公共就业保障服务体系；6、建设“中华健康快车白内障治疗中心”；7、推进教育创强工作，建成20个教育强镇(街)；8、继续更新公交车(含购进新能源汽车)80辆，建设候车亭一批。

各位代表!回顾过去，韶关人民艰苦奋斗，迎难而上，各项建设加快推进，改革发展开创了崭新局面。展望未来，我们实现科学发展蓝图的信心更加坚定。让我们更加紧密地团结在以胡锦涛同志为总书记的党中央周围，在市委的正确领导下，深入贯彻落实科学发展观，紧紧依靠全市人民，实事求是、解放思想、与时俱进，为推动经济社会跨越发展、建设幸福美好韶关而努力奋斗!
